# Supplementary material for: Incorporating physics to overcome data scarcity in predictive modeling of protein function: A case study of BK channels
Source: PLoS Comput Biol. 2023 Sep 15;19(9):e1011460. doi: 10.1371/journal.pcbi.1011460 (PMC10529646; doi:10.1371/journal.pcbi.1011460)
Supplement: S1 Text — The RMSF has the units of Å, and the mean percent SASA is a unitless percentage. These features were calculated from 100 ns MD simulation of the deactivated BK channel. Fig B. Correlation of experimental ∆V1/2 with the total Rosetta ∆∆∆G and LJ dispersion, the change in hydrophobicity upon mutation, and the Cα-Cα covariance with residue A316. The Pearson correlation coefficient is R < 0.01. See method section for description of how the Rosetta ∆∆∆G scores were calculated. Fig C. Distributions of (A) experimental ∆V1/2 values and (B) number of existing mutations along the sequence. Each histogram was generated using 100 bins. (A) depicts the ∆V1/2 distribution after squashing between ± 200 mV (see Methods). The key functional domains are highlighted beneath (B). Fig D. 5-fold cross-validation on initial training split. In all panels, blue denotes a data point used to train that iteration of the model, orange denotes validation or testing. The first five panels correspond to the 5-fold cross-validation within the training set (80% of the total data). The final panel (bottom right) was trained on the full training set and validated using the rest 20% of the full data set. This split corresponds to the 80/20 training/test split 1 from Table 1. Fig E. Feature Importance. Importance is reported as the mean decrease in Gini impurity score. A larger decrease in this impurity score means that using the feature in a branch of a tree often leads to greater ability to distinguish large and small shifts, for example. Names of features and their source are provided in Table B in S1 Text. Fig F. Performance of control model trained without physics-based descriptors. In all panels, blue dots denote data points used to train that iteration of the model, and orange dots denote the independent test data. Fig G. Correlation of the true and predicted error for the train and test sets of the five independent data splits. Blue and orange dot represent training and test data, respectively. [file pcbi.1011460.s001.pdf]

Supplementary Information

Incorporating physics to overcome data scarcity in predictive modeling of protein function: a case study of BK channels

Erik Nordquist[1,#], Guohui Zhang[2,#], Shrishti Barethiya[1], Nathan Ji[3], Kelli M. White[2], Lu Han[2], Zhiguang Jia[1], Jingyi Shi[2], Jianmin Cui[2], and Jianhan Chen[1,*]

[1] Department of Chemistry, University of Massachusetts Amherst, Amherst, Massachusetts, United States of America

[2] Department of Biomedical Engineering, Center for the Investigation of Membrane Excitability Disorders, Cardiac Bioelectricity and Arrhythmia Center, Washington University in St. Louis, St. Louis, Missouri, United States of America

[3] Department of Biology, Boston College, Chestnut Hill, Massachusetts, United States of America

[#] Co-first authors

* Corresponding author: jianhanc@umass.edu

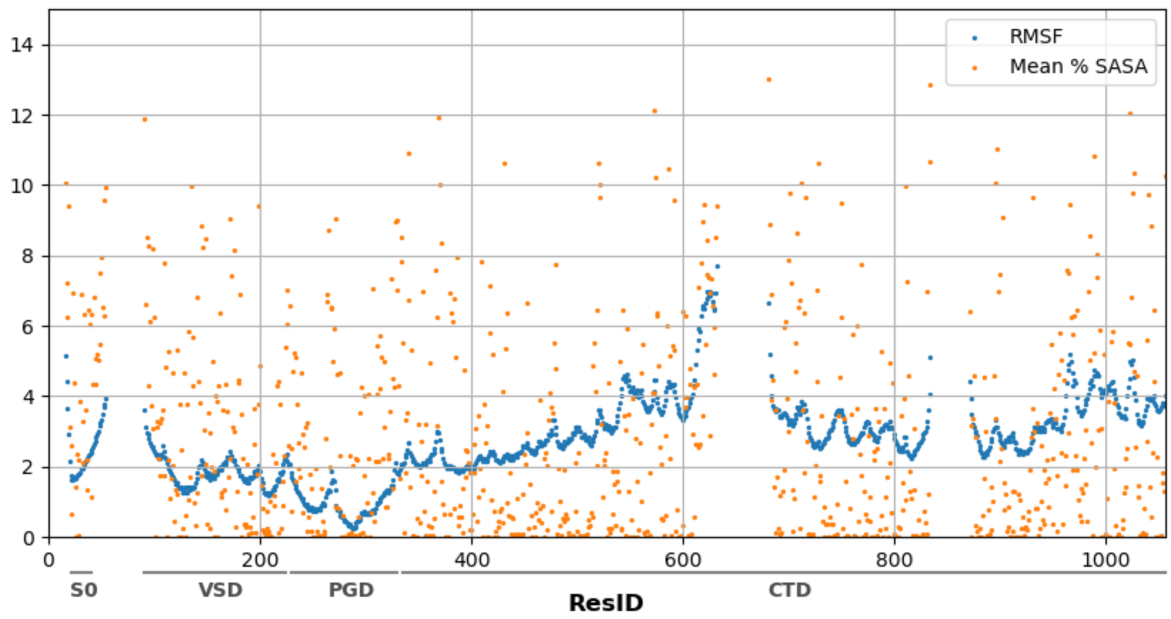

Fig A: Mean RMSF (blue) and percent SASA (orange) by Residue ID (ResID). The RMSF has the units of Å, and the mean percent SASA is a unitless percentage. These features were calculated from 100 ns MD simulation of the deactivated BK channel.

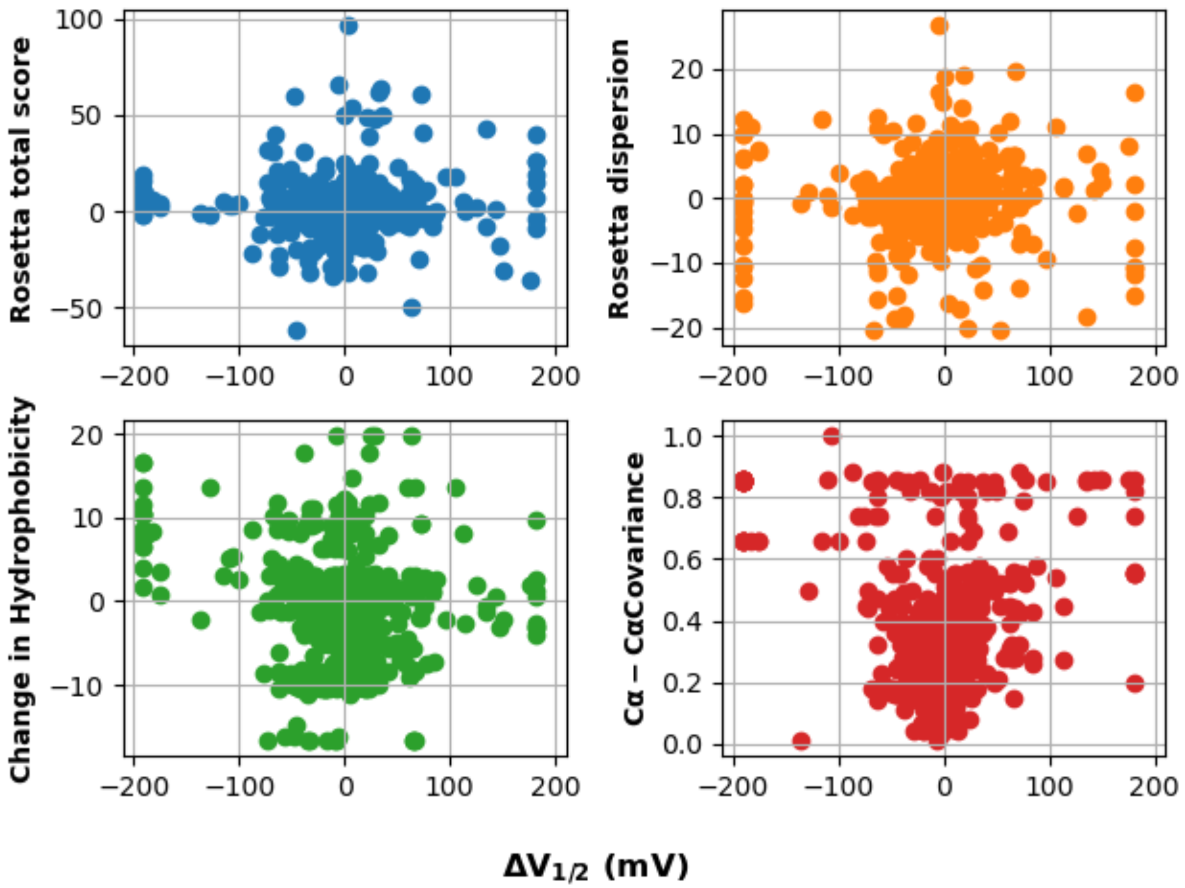

Fig B: Correlation of experimental $\Delta V_{1/2}$ with the total Rosetta ΔΔΔG and LJ dispersion, the change in hydrophobicity upon mutation, and the Cα-Cα covariance with residue A316. The Pearson correlation coefficient is $R < 0.01$ in all cases. See method section for description of how the Rosetta ΔΔΔG scores were calculated.

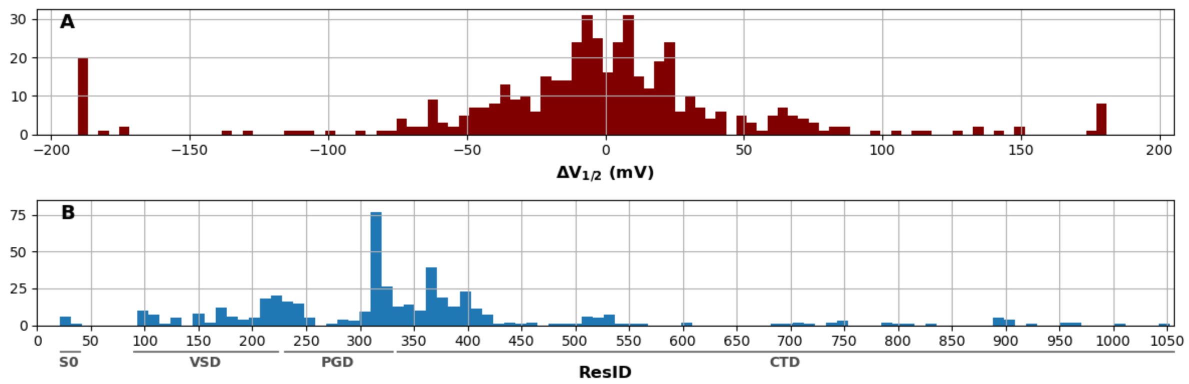

Fig C: Distributions of (A) experimental $\Delta V_{1/2}$ values and (B) number of existing mutations along the sequence. Each histogram was generated using 100 bins. **(A)** depicts the $\Delta V_{1/2}$ distribution after squashing between ± 200 mV (see Methods). The key functional domains are highlighted beneath **(B)**.

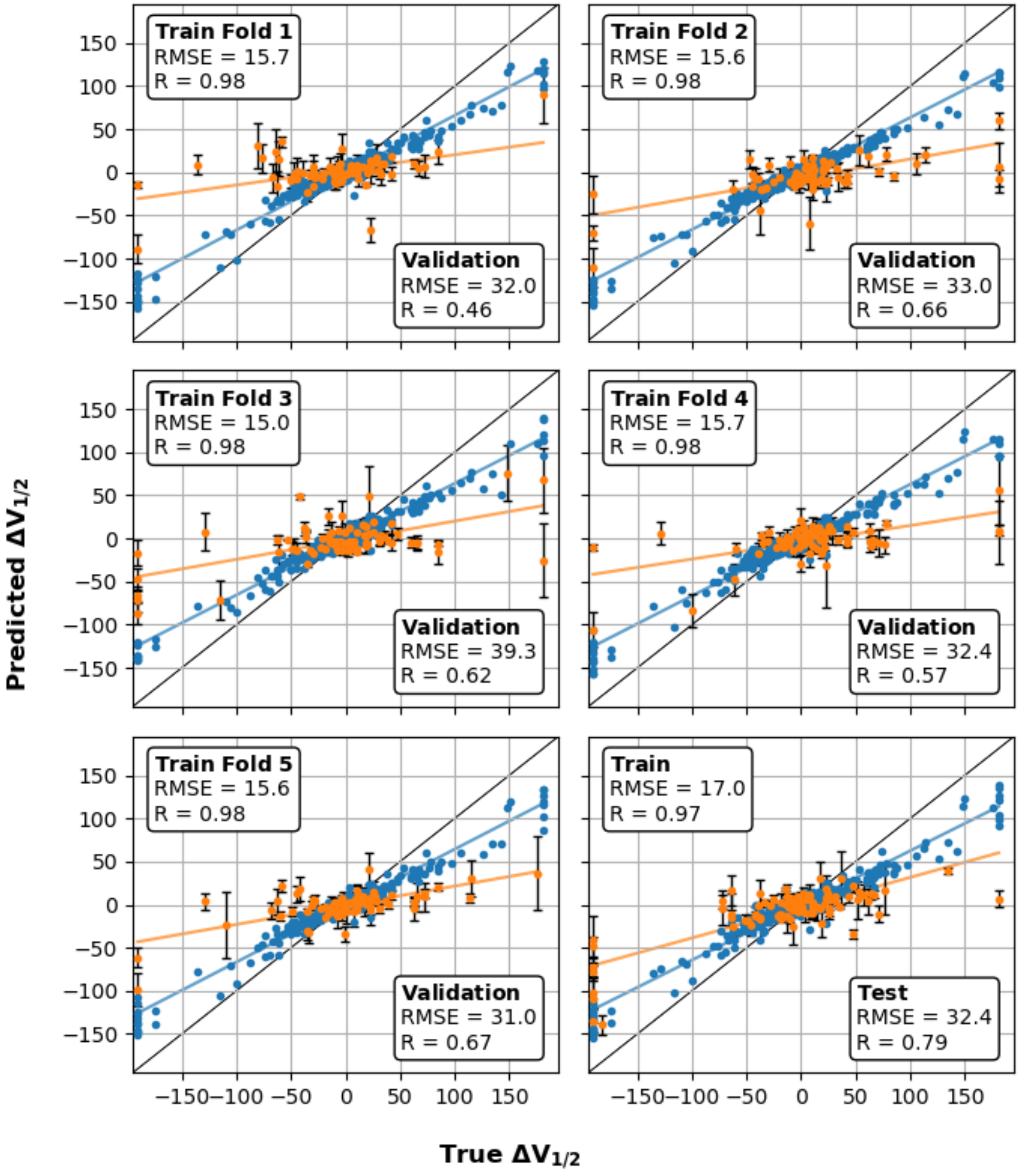

Fig D: 5-fold cross-validation on initial training split. In all panels, blue denotes a data point used to train that iteration of the model, orange denotes validation or testing. The first five panels correspond to the 5-fold cross-validation within the training set (80% of the total data). The final panel (bottom right) was trained on the full training set and validated using the rest 20% of the full data set. This split corresponds to the 80/20 training/test split 1 from Table 1.

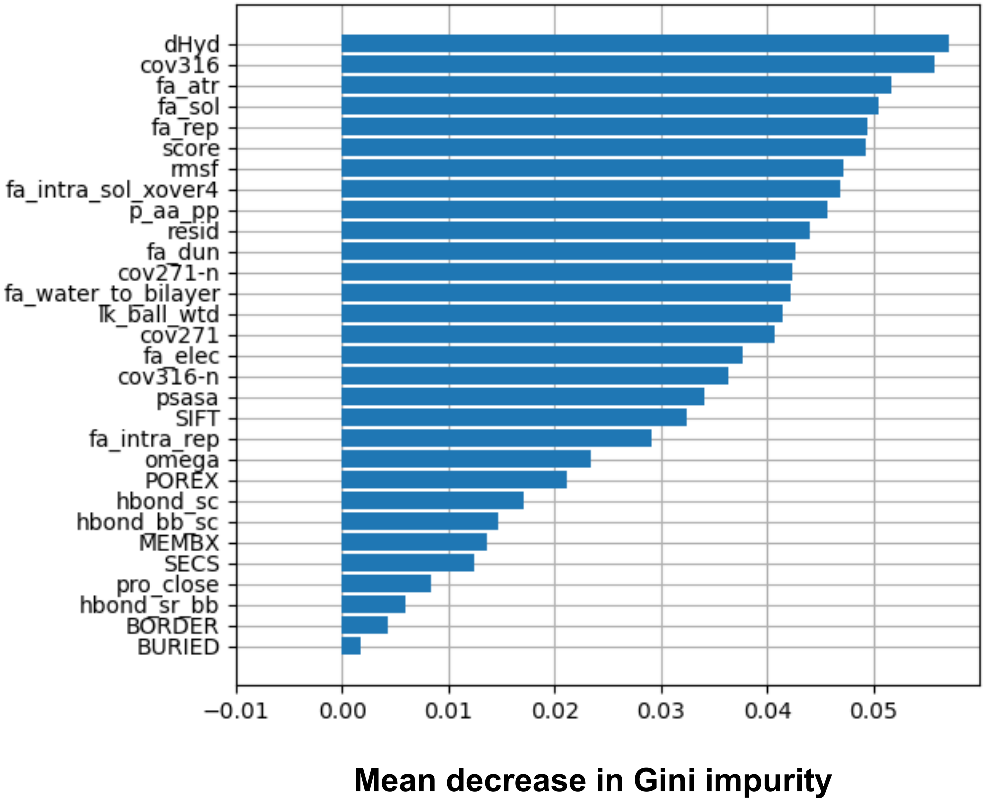

Fig E: Feature Importance. Importance is reported as the mean decrease in Gini impurity score. A larger decrease in this impurity score means that using the feature in a branch of a tree often leads to greater ability to resolve gating voltage shifts. Names of features and their source are provided in Table S2.

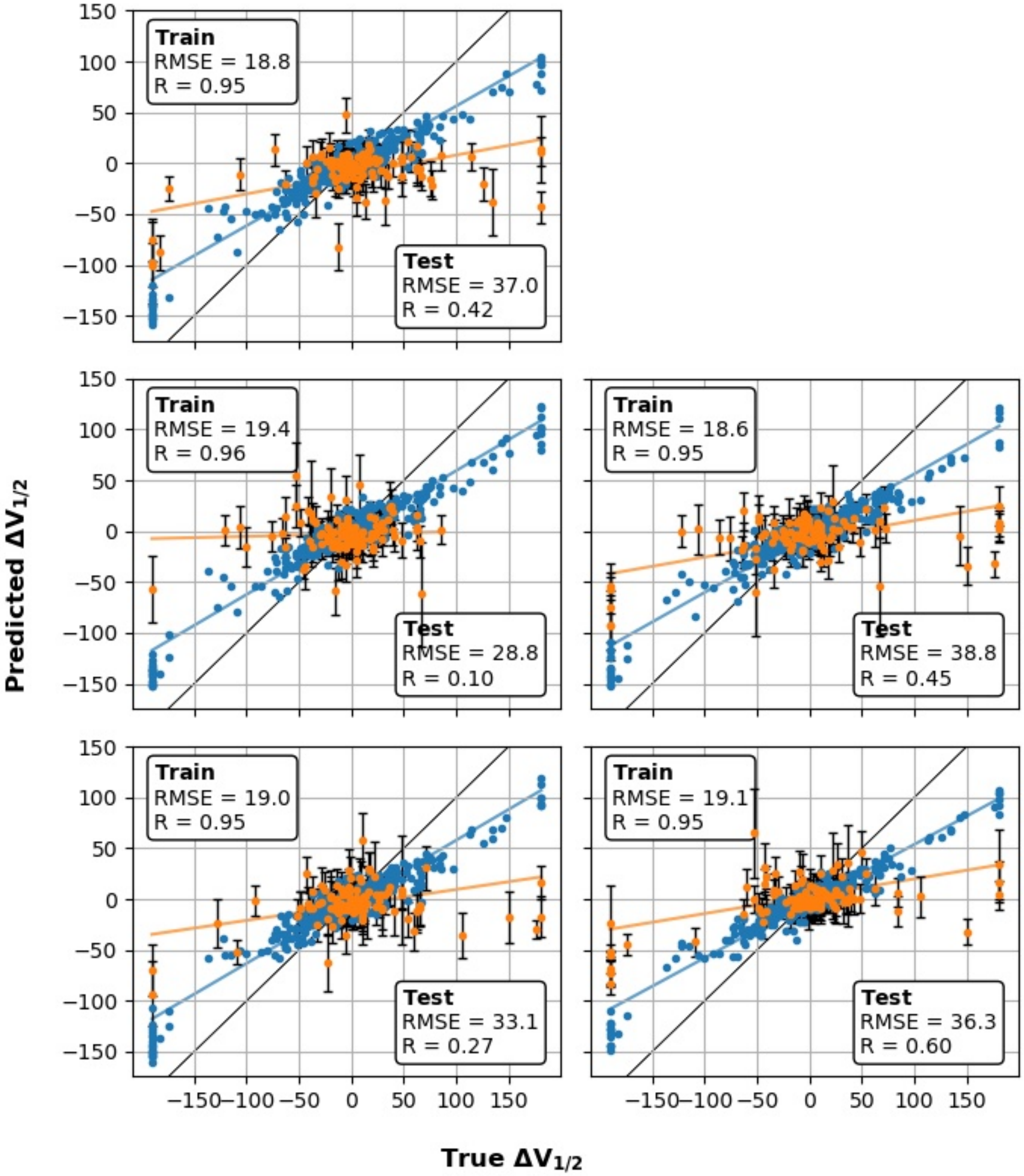

Fig F: Performance of control model trained without physics-based descriptors. In all panels, blue dots denote data points used to train that iteration of the model, and orange dots denote the independent test data.

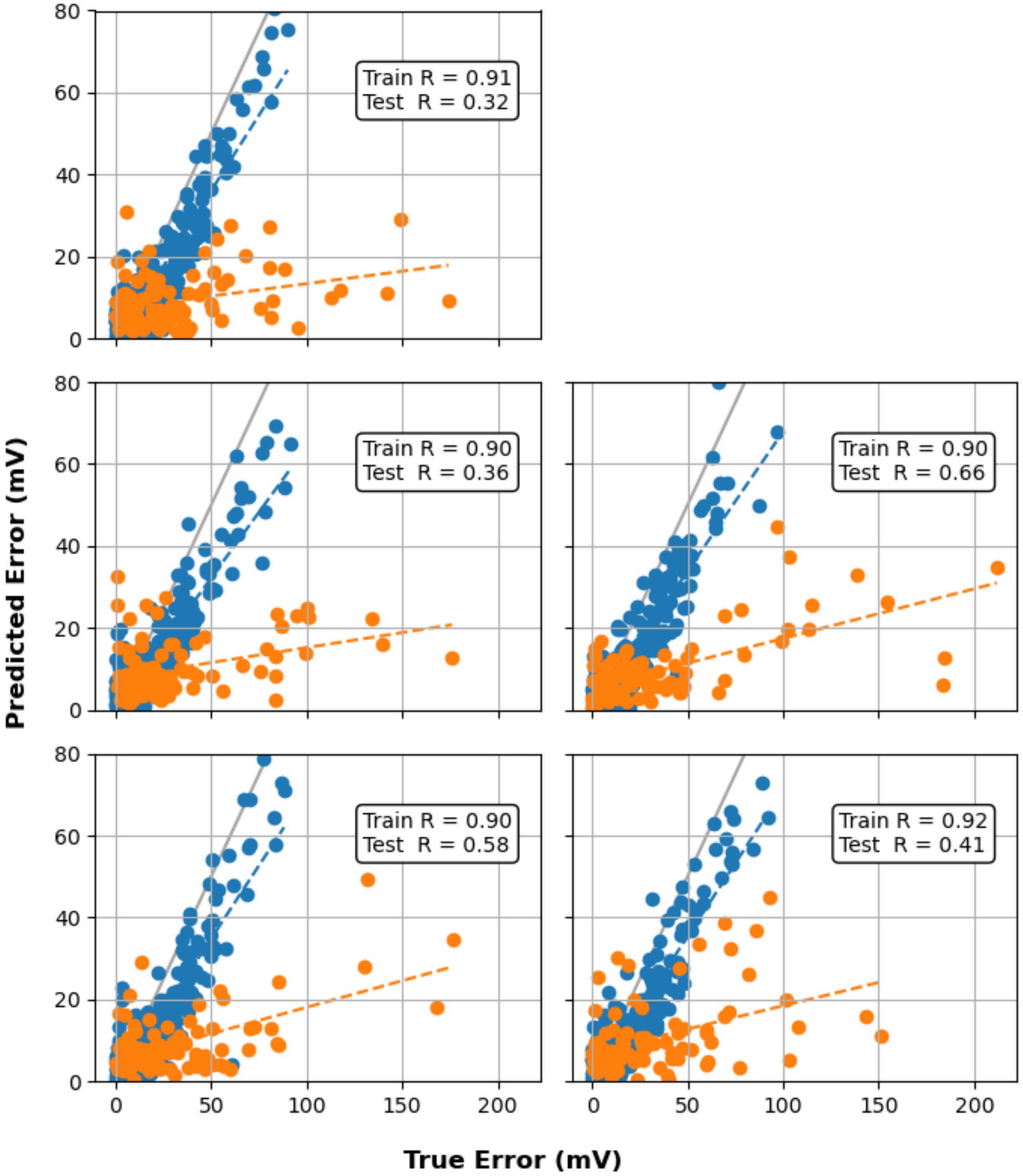

Fig G: Correlation of the true and predicted errors for the train and test sets of the five independent data splits. Blue and orange dot represent training and test data, respectively. The dashed lines denote lines of best fit with the same color scheme. The gray lines denote y=x.

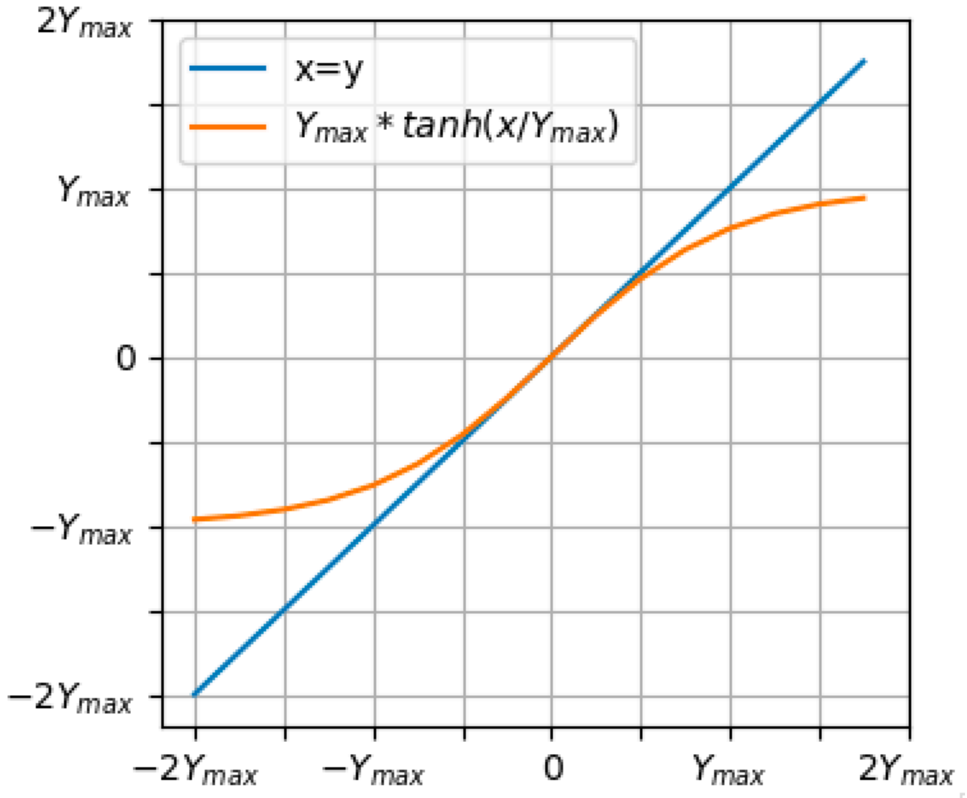

Fig H: Illustration of the squashing function for pre-processing of various raw quantities. The blue line plots the original, un-squashed function as a reference.

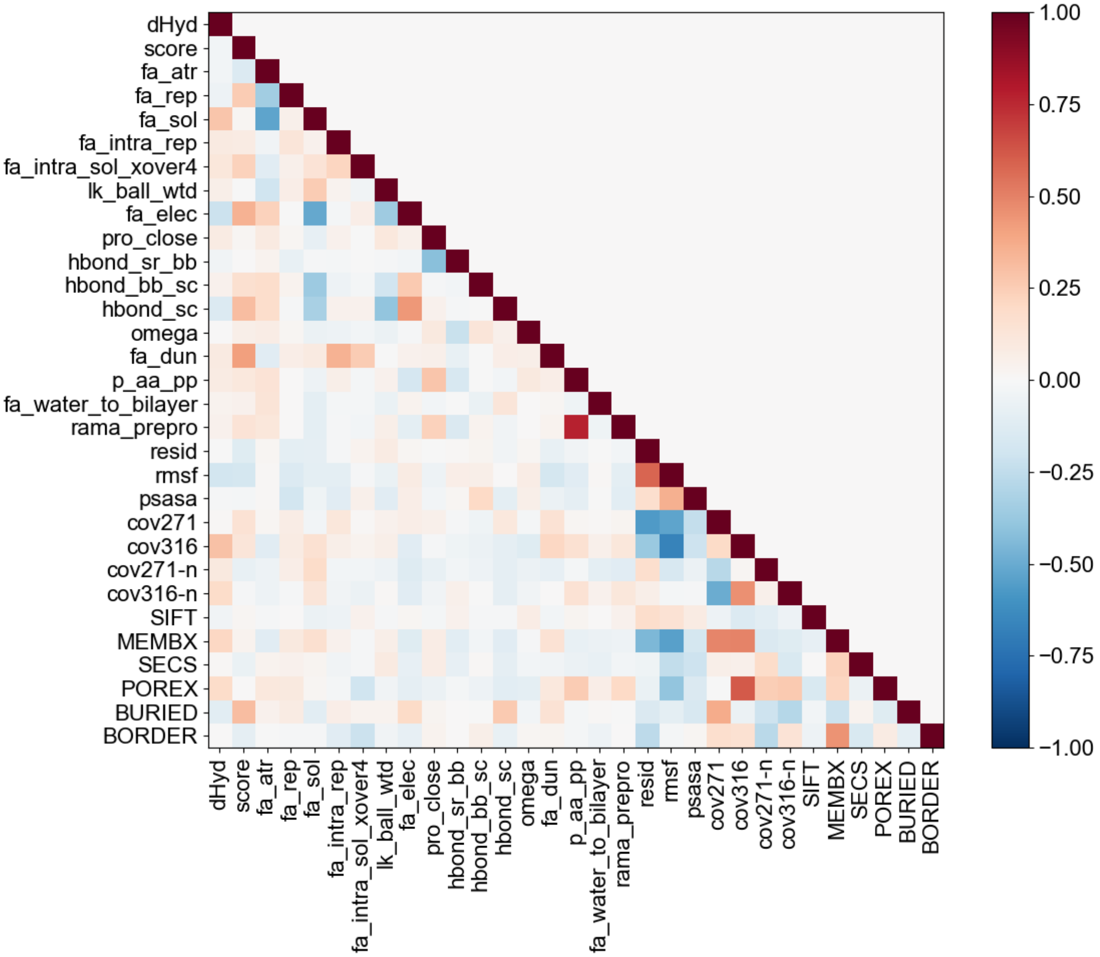

Fig I: Feature correlations. Correlation of the features used in the model training. Correlation is reported as the Pearson correlation coefficient. Names and descriptions of features and their sources are provided in Table S1.

Table A: List of features and their descriptions. All Rosetta terms are ΔΔΔG values between the closed and open states as described in Methods.

Feature#	Description#	Method
score	Total ΔG_{fold}	Rosetta ΔΔΔG
fa_atr	Lennard-Jones attractive between atoms in different residues.	Rosetta ΔΔΔG
fa_rep	Lennard-Jones repulsive between atoms in different residues.	Rosetta ΔΔΔG
fa_sol	Lazaridis-Karplus solvation energy.	Rosetta ΔΔΔG
fa_intra_rep	Lennard-Jones repulsive between atoms in the same residue.	Rosetta ΔΔΔG
fa_elec	Coulombic electrostatic potential with a distance-dependent dielectric.	Rosetta ΔΔΔG
pro_close	Proline ring closure energy and energy of Ψ angle of preceding residue.	Rosetta ΔΔΔG
hbond_sr_bb	Backbone-backbone hydrogen bonds close in primary sequence.	Rosetta ΔΔΔG
hbond_lr_bb	Backbone-backbone hydrogen bonds distant in primary sequence.	Rosetta ΔΔΔG
hbond_bb_sc	Sidechain-backbone hydrogen bond energy.	Rosetta ΔΔΔG
hbond_sc	Sidechain-sidechain hydrogen bond energy.	Rosetta ΔΔΔG
dslf_fa13	Disulfide geometry potential.	Rosetta ΔΔΔG
rama	Ramachandran preferences.	Rosetta ΔΔΔG
omega	A harmonic restraint on planarity of the ω backbone dihedral with standard deviation of ~6°.	Rosetta ΔΔΔG
fa_dun	Internal energy of sidechain rotamers as derived from Dunbrack's rotamer statistics (2010 Rotamer Library used in Talaris2013).	Rosetta ΔΔΔG
p_aa_pp	Probability of amino acid at Φ/Ψ.	Rosetta ΔΔΔG
yhh_planarity	A special torsional potential to keep the tyrosine hydroxyl in the plane of the aromatic ring.	Rosetta ΔΔΔG
ref	Reference energy for each amino acid that balances the internal energy of amino acid terms.	Rosetta ΔΔΔG
fa_intra_sol_xover4	Intra-residue LK solvation, counted for the atom-pairs beyond torsion-relationship.	Rosetta ΔΔΔG
fa_intra_elec	Intra-residue Coulombic interaction, counted for the atom-pairs beyond torsion-relationship.	Rosetta ΔΔΔG
rama_prepro	Backbone torsion preference term that accounts for whether preceding amino acid is Proline or not.	Rosetta ΔΔΔG
lk_ball	Anisotropic contribution to the solvation.	Rosetta ΔΔΔG

cov316	Row of intra-monomer Ca-Ca covariance matrix corresponding to residue A316, describes coupled motion with the pore	MD
cov278	Row of intra-monomer Ca-Ca covariance matrix corresponding to residue X278, 278 also has several mutations with large shifts and is not covariant with the pore	MD
cov316-n	Row of directly-neighboring inter-monomer Ca-Ca covariance matrix corresponding to residue A316, describes coupled motion with the pore	MD
cov278-n	Row of directly-neighboring inter-monomer Ca-Ca covariance matrix corresponding to residue X278, 278 also has several mutations with large shifts and is not covariant with the pore	MD
RMSF	Root-mean square fluctuations, describes chain flexibility	MD
pSASA	Percent Solvent-Accessible Surface Area, describes solvation effects including in the deep pore.	MD
dHyd	Change in hydrophobicity from wildtype to mutant. Hydrophobicity is transfer free energy from cyclohexane to water.	Biochemical data (1)
ResID	The residue ID or number, following the numbering scheme of the PDB entries.	--
WATX	Residue is within 5 Å of water in equilibrated snapshot	MD
PORX	Residue is pore-lining	Structure
MEMX	Residue is within 5 Å of lipid in equilibrated snapshot	Structure
BORDER	Both WATX and MEMX, residue is at membrane interface	MD/Structure
SECS	Secondary Structure (helix, coil, sheet)	Structure
SIFT	Score from the SIFT algorithm: Sorting Intolerant from Tolerant, predict effect of single nucleotide polymorphism based partially on sequence conservation score	SIFT tool (2)

Based on: https://www.rosettacommons.org/docs/latest/rosetta_basics/scoring/score-types and https://www.ncbi.nlm.nih.gov/pmc/articles/PMC5717763/table/T1/

Table B: Summary of machine learning models, hyperparameters trained in Grid Search, and training and validation correlation (R) and RMSE from 5 independent splits of data.

Model	Hyperparameters	R<br>(Train, Test)	RMSE (mV)<br>(Train, Test)
Ridge	**alpha**: 1e-6, 1e-4, 1e-2, 1, 10, **1e2**, 1e4, 1e6	(0.40, 0.33),<br>(0.39, 0.29),<br>(0.41, 0.21),<br>(0.39, 0.22),<br>(0.41, 0.24)	(37, 44),<br>(39, 37),<br>(37, 44),<br>(37, 44),<br>(38, 42)
SVR	**kernel**: 'linear', 'poly', 'sigmoid', **'rbf'**;<br>**degree**: 2, **3**, 6;<br>**C**: 1e-2, 1e-1, 1, 1e1, **1e2**;<br>**coef0**: 0, **1**;<br>**epsilon**: 0, 1e-4, 1e-2, **1**, 1e2	(0.81, 0.69),<br>(0.83, 0.52),<br>(0.83, 0.57),<br>(0.80, 0.60),<br>(0.82, 0.57)	(19, 35),<br>(18, 32),<br>(18, 36),<br>(18, 34),<br>(18, 35)
RF	**n_estimators**: 50, 100, 250, **500**, 1000;<br>**min_samples_split**: **2**, 5, 10, 20;<br>**max_leaf_nodes**: 2, 5, 10, 20, 50, **100**, 200;<br>**max_depth**: 2, 5, 10, **20**, 50, 100;<br>**max_features**: 0.1, 0.25, 0.5, 0.75, 0.9, 1.0;<br>**ccp_alpha**: 1e-3, **1e-2**, 1e-1, 1, 10, 100;<br>**max_samples**: 0.1, 0.25, 0.5, 0.75, **1.0**;<br>**min_samples_leaf**: **1**, 2, 5, 10, 20;<br>**min_weight_fraction_leaf**: **0**, 0.01, 0.1, 0.25, 0.5;	(0.97, 0.79),<br>(0.97, 0.54),<br>(0.97, 0.69),<br>(0.97, 0.80),<br>(0.98, 0.70)	(17, 32),<br>(17, 30),<br>(16, 35),<br>(17, 31),<br>(16, 31)
KNN	**n_neighbors**: **3**, 7, 11, 21, 51, 101;<br>**weights**: **'uniform'**, 'distance';<br>**leaf_size**: **5**, 10, 50, 100;<br>**p**: **1**, 2, 3;<br>**algorithm**: 'ball_tree', **'kd_tree'**, 'brute'	(0.81, 0.73),<br>(0.84, 0.62),<br>(0.83, 0.77),<br>(0.82, 0.63),<br>(0.82, 0.52)	(23, 30),<br>(21, 29),<br>(22, 29),<br>(23, 33),<br>(22, 34)
GP	**n_estimators**: 10, **50**, 100, 500, 1000;<br>**max_depth**: 2, 4, 16, **32**, 64;<br>**min_samples_split**: 2, 4, 16, 32, 64;<br>**max_features**: 0.25, 0.5, **0.75**, 1.0;<br>**ccp_alpha**: 0, **5e-3**, 10e-3, 20e-3	(0.92, 0.57),<br>(0.87, 0.30),<br>(0.87, 0.54),<br>(0.92, 0.62),<br>(0.87, 0.64)	(34, 41),<br>(36, 34),<br>(34, 42),<br>(35, 39),<br>(35, 38)
MLP	**solver**: 'sgd'; **learning_rate**: invscaling;<br>**power_**t: 1e-6, 1e-4, **1e-2**, 1;<br>**activation**: 'tanh';<br>**hidden_layer_sizes**: (10), (50), **(100)**, (500), (100,10,100);<br>**alpha**: 1e-6, 1e-4, 1e-2, **1**	(1.0, 0.77),<br>(1.0, 0.40),<br>(1.0, 0.76),<br>(1.0, 0.74),<br>(1.0, 0.66)	(2, 30),<br>(2, 37),<br>(2, 31),<br>(2, 31),<br>(2, 35)

Table C: Neurological BK channel mutants, channel activity phenotypes, functional mechanisms (if known), and predicted $\Delta V_{1/2}$ values. Abbreviations: VUS: Variant of Uncertain Significance, NE: No effect, LOF: Loss of Function, and GOF: Gain of Function. The Coordination of Rare Diseases at Sanford (CoRDS) is a standardized patient registry in a de-identified format. NP stands for No Prediction; the model doesn't predict any shift for mutations to residues absent in either the Ca^{2+}-bound or unbound PDB structure.

PDB Mutation Name	Mutation Name (hslo1 gene)	BK channel activity	Functional Mechanism	Predicted $\Delta V_{1/2}$ (mV)	Reference
	G20D	VUS		NP	(3)
	I29F	VUS		NP	(3)
L206F	L271F	VUS		19 ± 14	(3)
S286Y	S351Y	LOF	No current at 160 mV and 10 μM Ca^{2+}.	14 ± 3	(4)
G289S	G354S	LOF	G-V shift to depolarized potentials, restored by NS1619	5 ± 13	(5)
G291R	G356R	LOF	No current at 160 mV and 10 μM Ca^{2+}.	-9 ± 12	(4)
G310R	G375R	LOF	No Current	-2 ±7	(4)
C348Y	C413Y	LOF	G-V shift to depolarized potentials, decreased expression	5 ± 9	(4)
D369G	D434G	GOF	G-V shift to hyperpolarized potentials, increased open probability, faster activation, slower deactivation, increased Ca^{2+} sensitivity	-13 ± 4	(5)
H379Q	H444Q	LOF		3 ± 4	(6), CoRDS
K392E	K457E	LOF	G-V shift to depolarized potentials	32 ± 12	(7)
I447V	I512V	VUS		-2 ± 2	(8), CoRDS
K453N	K518N	NE		-9 ± 1	(9)
A467V	A532V	VUS		-7 ± 4	CoRDS

N471H	N536H	GOF		-8 ± 5	(10)
E500A	E565A	NE		NP	(9)
G502S	G567S	VUS		-3 ± 4	CoRDS
E591A	E656A	VUS		NP	(9)
I598V	I663V	LOF		3 ± 3	(4)
E671K	E736K	VUS		NP	(3)
D735Y	D800Y	VUS		NP	CoRDS
P740L	P805L	LOF	G-V shift to depolarized potentials, decreased expression	-17 ± 5	(4)
E819K	E884K	NE		4 ± 13	(11)
D900V	D965V	LOF		-7 ± 6	(3, 6)
D919N	D984N	LOF		NP	(4)
N988S	N1053S	GOF	G-V shift to hyperpolarized potentials, increased open probability, faster activation, slower deactivation, Ca^{2+}-independent mechanism	NP	(3), CoRDS
R1018K	R1083K	VUS		-13 ± 5	(12)
R1032H	R1097H	LOF		NP	(6)
T1046R	T1111R	VUS		-6 ± 7	(3)
R1063W	R1128W	NE		NP	(13)
T1089I	T1154I	VUS		NP	(3)
N1094S	N1159S	NE		NP	(9)

SI References

1. A. Radzicka, R. Wolfenden, Comparing the polarities of the amino acids: side-chain distribution coefficients between the vapor phase, cyclohexane, 1-octanol, and neutral aqueous solution. *Biochemistry* **27**, 1664–1670 (1988).

2. P. C. Ng, SIFT: predicting amino acid changes that affect protein function. *Nucleic Acids Research* **31**, 3812–3814 (2003).

3. J. P. Miller, H. J. Moldenhauer, S. Keros, A. L. Meredith, An emerging spectrum of variants and clinical features in KCNMA1-linked channelopathy. *Channels* **15**, 447–464 (2021).

4. L. Liang, *et al.*, De novo loss-of-function KCNMA1 variants are associated with a new multiple malformation syndrome and a broad spectrum of developmental and neurological phenotypes. *Hum Mol Genet* **28**, 2937–2951 (2019).

5. W. Du, *et al.*, Calcium-sensitive potassium channelopathy in human epilepsy and paroxysmal movement disorder. *Nat Genet* **37**, 733–738 (2005).

6. H. J. Moldenhauer, S. Mi Park, A. L. Meredith, Characterization of New Human KCNMA1 Loss-of-function Mutations. *Biophysical Journal* **118**, 114a (2020).

7. C. Buckley, *et al.*, Status Dystonicus, Oculogyric Crisis and Paroxysmal Dyskinesia in a 25 Year-Old Woman with a Novel KCNMA1 Variant, K457E. *Tremor Other Hyperkinet Mov (N Y)* **10**, 49.

8. W.-T. Tian, *et al.*, Proline-rich transmembrane protein 2-negative paroxysmal kinesigenic dyskinesia: Clinical and genetic analyses of 163 patients. *Mov Disord* **33**, 459–467 (2018).

9. X. Li, *et al.*, De novo BK channel variant causes epilepsy by affecting voltage gating but not Ca2+ sensitivity. *Eur J Hum Genet* **26**, 220–229 (2018).

10. G. Zhang, *et al.*, A Gain-of-Function Mutation in KCNMA1 Causes Dystonia Spells Controlled With Stimulant Therapy. *Mov Disord* **35**, 1868–1873 (2020).

11. Z.-B. Zhang, M.-Q. Tian, K. Gao, Y.-W. Jiang, Y. Wu, De novo KCNMA1 mutations in children with early-onset paroxysmal dyskinesia and developmental delay. *Movement Disorders* **30**, 1290–1292 (2015).

12. H. Wu, *et al.*, Phenotype-to-genotype approach reveals head-circumference-associated genes in an autism spectrum disorder cohort. *Clin Genet* **97**, 338–346 (2020).

13. H. J. Moldenhauer, K. K. Matychak, A. L. Meredith, Comparative gain-of-function effects of the *KCNMA1* -N999S mutation on human BK channel properties. *Journal of Neurophysiology* **123**, 560–570 (2020).
