# Supplementary material for: Incorporating physics to overcome data scarcity in predictive modeling of protein function: A case study of BK channels
Source: PLoS Comput Biol. 2023 Sep 15;19(9):e1011460. doi: 10.1371/journal.pcbi.1011460 (PMC10529646; doi:10.1371/journal.pcbi.1011460)
Supplement: Attachment — Submitted filename: Response2Reviewer.docx [file pcbi.1011460.s002.docx]

**Note:** the original comments are quoted in italic below.

*Reviewer #1:*

*The manuscript presents a study combining physically motivated molecular features with sparse biochemical data to predict biomolecule functional transitions. This is the critical challenge that much of the molecular biophysics community faces nowadays. The authors show one interesting approach to dealing with such a challenge using ion channel gating. Using simulations and sparse but still significant experimental data, authors train a random forest model to predict gating trends. The model shows robustness in predicting global trends (hydrophobic nature of gating) and shows an impressive ability even to predict some subtle mutations on the helix of the transmembrane region.*

*That being said, I found some parts of the manuscript confusing and lacking in crucial details, preventing me from fully understanding some of the authors' choices with their methodology. Specifically, the following points can be improved:*

**Author response:** We thank the reviewer for the support and thoughtful comments.

*The description of the dataset and all the considered features used in training must be motivated and listed early on in the results section. Having a section dedicated to (i) carefully describing the dataset, (ii) containing a table listing names and variables extracted from MD simulations, rosetta calculations, and (iii) references and to databases and papers from where data is collected, (iv) visualization of the dataset correlated with simple features to allow readers to see what the challenge is.*

**Author response:** We agree that it is important to fully describe all features used in training. In the revised manuscript, we have now clarified all the four points raised and made appropriate changes to the manuscript.

(i) The physics-based features are described in detail and carefully motivated in first Results section “*Physics-based features …*” (**Page 6**). Visualization of the dataset is shown in **Figure 2**. We have added discussion of the distribution of the dataset and moved the first mention of **Figure S3** to the beginning of the second Results section “*Random forest …*” (**page 8**). We have discussed the experimental dataset in the opening lines of the same section and in greater detail first **Methods** section (**page 18**).
(ii) We have included a table in the SI (**Table S1**) which lists all the features by name and abbreviation extracted from MD, Rosetta, etc… and a description of each. We feel that this table belongs in the SI.
(iii) We have **included as a supplemental material available on Github** the excel file containing all mutations, there source(s), and any notes about the measurement where necessary. We did not include this file in the initial submission, but it is **referenced in the text on page 8, first paragraph (opening sentence).**
(iv) We have **three new panels to the supplemental Figure S2** in addition to the Rosetta dddG score, namely the additional features are the change in hydrophobicity upon mutation, Rosetta LJ dispersion term, and Ca-Ca Covariance with Resid 316. These panels illustrate a lack of strong correlation of any input features with dV1/2.

*The paper needs to describe the choice and nature of training and test splitting choice better. These should be carefully motivated. Have authors considered various forms of scaling of heterogeneous features? This is often decisive in sparse datasets with magnitudes of features on vastly different scales.*

**Author response:** This is indeed an important aspect of the training. We have clarified that the 80/20 splits were performed randomly using the train_test_split function provided in the python machine learning library scikit-learn (see **page 20,** under **“***Statistical model selection, training and validation***”**). Furthermore, we agree that feature scaling is an important aspect of performance. We stated on **Page 20** that “*We then standardized the remaining features to have a mean of zero and unit variance* …”.

*Fig 2 I found it could have been more useful for reinforcing the main results. Could authors plot the effects of mutations on voltage on a plot and highlight train and test sets? or show a correlation with the degree of hydrophobicity.*

**Author response:** Thank you for this comment. As discussed above, we have expanded the Figure S2 to include the correlation of four features, including the change in hydrophobicity, against the experimental dV1/2.

*The authors looked at Ca-Ca distances, a rather coarse measure for using in detailed atomistic effects such as changes in side chains introduced in mutations. Other atomically detailed measures could be considered, such as the number of hydrophobic clusters, hydrogen bonding networks, or salt bridges.*

**Author response:** Indeed, we only included Ca-Ca covariance, RMSF and SASA from our atomistic simulations but additional atomistic effects could be included as suggested. The rationale for not including these features is that their importance to the open-close equilibrium of the channel is already quantified in Rosetta energetic analysis. In particular, the physical features suggested by the reviewer are already captured in various Rosetta terms, including the effects of hydrophobic interactions (LJ dispersion term), hydrogen bonding (explicitly) and salt bridges (electrostatics term).

In the Results section on **page 6**, we state “*These individual terms quantify the contributions of different physical interactions and thus provide useful additional information for training the predictive mode*.”

*In the results sections, authors could place their work in the context of prior studies by reviewing the literature on using RF/ML to predict functional mutations.*

*Chen, A.S.Y., Westwood, N.J., Brear, P., Rogers, G.W., Mavridis, L. and Mitchell, J.B., 2016. A random forest model for predicting allosteric and functional sites on proteins. Molecular informatics, 35(3‐4), pp.125-135.*

*Rodrigues CH, Pires DE, Ascher DB. DynaMut: predicting the impact of mutations on protein conformation, flexibility and stability. Nucleic acids research. 2018 Jul 2;46(W1):W350-5.*

*Dutagaci B, Duan B, Qiu C, Kaplan CD, Feig M. Characterization of RNA polymerase II trigger loop mutations using molecular dynamics simulations and machine learning. PLOS Computational Biology. 2023 Mar 22;19(3):e1010999*

*Yasuda I, Endo K, Hirano Y, Yamamoto E, Yasuoka K. Relation in ligand dynamics and protein mutation using unsupervised machine learning. Biophysical Journal. 2023 Feb 10;122(3):182a.*

**Author response:** Thank you for pointing out this out. We have added these references, as well as another related paper (Phul, et al. 2022 PLoS Comp Biol.), to our discussion of the incorporation of physics-based data in machine learning in the Introduction (**new references 58-62; page 4, first paragraph**)

*Reviewer #2:*

*The authors present an interesting work where they incorporated predicted physical properties from MD simulations to an existing data set of protein mutation, to build a predictive model of protein function. They used the BK channels as a case study for their proposed method.

Overall, the idea of extending existing data set with simulation data is appealing, and it is reasonable to expect it to improve the model predictive ability. Focusing this review on the modeling effort, I have a few questions:*

**Author response:** We thank you for your supportive and constructive comments.

*1. I'm unfamiliar with how the mutation data is usually represented, and I don't see it anywhere explained in the paper. The author could include a brief description of this data and how it is incorporated in the ML model. The topic is interesting and adding this information may extend the readability of the paper for readers interested in combining data and simulation results.*

**Author response:** The experimental mutational data is the ∆V1/2 for each single mutation. It is not represented in the model directly, but by training the model’s physics-based features describing the impact of the mutation (discussed in detail in both the early Results section and the Methods) to reproduce the known experimental ∆V1/2. We have discussed the experimental dataset and data collection in the Methods sections “Electrophysiology data collection and preparation” (**page 18**).

*2. The authors use the simulation data to extend the dimensionality of the input data X for training the model, without increasing the size of the data set. That in itself would not be a problem, if the original data set covered a larger fraction of possible mutations. When commenting about the performance of the model over the different splits, the authors claim that the model perform better when training and test set have similar coverage. Which raises the question, if we are covering only ~3% of your mutation space, and the coverage seems to be very important in the performance of the model. How can this model be generalized to unseen mutations.*

**Author response:** Thank you for this excellent point about the caveats of using the model across the complete sequence. We have discussed the limitations of the model in the Results section (**page 12, both paragraphs**) as well as the Conclusions (**page 17, second paragraph**). We further note in the revised manuscript that “*It should be noted that both the hydrophobic gating and effect of L235/V236 mutations are localized to the PGD, the region with the best experimental coverage*” (**page 17**)

*In the same line, it would be interesting to see how the mutations are encoded in the data, and what coverage means exactly in this context.*

**Author response:** We have discussed how the effects of mutations are described in our physics-based features in the Results section title “Physics-based features…” (**page 6**) and several Methods sections (**page 19-21**). The coverage of experimental data is illustrated in **Figure 1** as well as supplementary **Figure S3**.

3. Please, verify the sue of RMSE and RMSF

**Author response:** We have checked that the references to RMSE (root-mean square error) and RMSF (root-mean square fluctuation) are correct, respectively, and moved the definition of the acronym to be the first appearance of the acronym in the main text. Previously they had been in the methods section.
